# CXCL8 in Tumor Biology and Its Implications for Clinical Translation

**DOI:** 10.3389/fmolb.2022.723846

**Published:** 2022-03-15

**Authors:** Xingyu Xiong, Xinyang Liao, Shi Qiu, Hang Xu, Shiyu Zhang, Sheng Wang, Jianzhong Ai, Lu Yang

**Affiliations:** ^1^ Department of Urology, National Clinical Research Center for Geriatrics, Institute of Urology, West China Hospital of Sichuan University, Chengdu, China; ^2^ Center of Biomedical Big Data, West China Hospital, Sichuan University, Chengdu, China; ^3^ Department of Endocrinology and Metabolism, West China Hospital of Sichuan University, Chengdu, China

**Keywords:** CXCL8, tumor microenvironment, tumor progression, tumor immune suppression, immunotherapy

## Abstract

The chemokine CXCL8 has been found to play an important role in tumor progression in recent years. CXCL8 activates multiple intracellular signaling pathways by binding to its receptors (CXCR1/2), and plays dual pro-tumorigenic roles in the tumor microenvironment (TME) including directly promoting tumor survival and affecting components of TME to indirectly facilitate tumor progression, which include facilitating tumor cell proliferation and epithelial-to-mesenchymal transition (EMT), pro-angiogenesis, and inhibit anti-tumor immunity. More recently, clinical trials indicate that CXCL8 can act as an independently predictive biomarker in patients receiving immune checkpoint inhibitions (ICIs) therapy. Preclinical studies also suggest that combined CXCL8 blockade and ICIs therapy can enhance the anti-tumor efficacy, and several clinical trials are being conducted to evaluate this therapy modality.

## Introduction

The chemokine CXCL8, also known as interleukin-8 (IL-8), is initially known as a cytokine expressed by epithelial cells and macrophages for neutrophil recruitment to areas of inflammation, infection, or injury (Horn et al., 2020a). The biological effects of CXCL8 are mediated through its binding to two cell-surface G-protein-coupled receptors: CXCR1 and CXCR2, which are generally expressed on monocytes, granulocytes, and endothelial cells (Waugh and Wilson, 2008; Liu et al., 2016). Furthermore, CXCL8 monomer binds CXCR1 with high affinity, however, both monomer and dimer show similar affinities to CXCR2 (Helen et al., 2017).

Although CXCL8 has been originally described as a proinflammatory chemokine, in the context of cancer, CXCL8 is produced by multiple cell types in the tumor microenvironment (TME), including the infiltrating immune cells, stromal cells, and the tumour cells (Waugh and Wilson, 2008; Alfaro et al., 2017). Additionally, the mechanism of CXCL8-CXCR1/2 pathway in tumourigenesis, tumour progression and immune suppression in TME has been explored extensively. Recent investigations demonstrate several novel mechanisms of the crosstalk between CXCL8 and components in TME to facilitate tumor progression, even forming positive feedback loops. Immune checkpoint inhibitions (ICIs) have become the cornerstone of immunotherapy in many types of cancers. Emerging trials underline the crucial roles of CXCL8 in ICIs therapy.

In this review, we summarized the current understanding of CXCL8 signaling cascades and recently developed mechanisms of facilitating tumor survival, invasion, and immune suppression. Additionally, we discussed the CXCL8 as a biomarker of ICIs therapy and the role of anti-CXCL8 as a combination agent in immunotherapy.

## Structure and Secretion of CXCL8

CXCL8 is initially translated as a protein with 99 amino acids, which is subsequently processed into two active isoforms: 1) 72 amino acids in monocytes and macrophages; 2) 77 amino acids in non-immune cells (Waugh and Wilson, 2008). According to the position of the first two cysteine residues on the N-terminus, chemokines can be divided into four highly conserved subtypes: CXC (the two cysteines nearest the N-termini are separated by a another single amino acid), CC (the first two cysteines nearest the N-termini are adjacent), C (only one cysteine near its N-terminus) and CX3C (with three amino acids between the first two cysteines at the N-terminal) (Rollins, 1997; Balkwill, 2004). Further, the family of CXC chemokines can be divided into to ELR- and ELR + groups based on the absence or presence of the tripeptide Glu-Leu-Arg (the ELR motif) which precedes the cysteine on the N-terminus (Baggiolini et al., 1997). CXCL8 is one of the ELR + CXC chemokines (Baldwin et al., 1991).

The gene encoding CXCL8 is located on 4q13-q21 (Modi et al., 1990). There are four common polymorphisms in the CXCL8 gene: rs4073(-251 A/T), rs2227532(-845T/C), rs2227307(+396 G/T) and rs2227306(+781 C/T) (Mukaida et al., 1989; Yao et al., 2019). Previously studies indicated that the single nucleotide polymorphism (SNPs) of CXCL8 gene were significantly associated with increased risk or progression of non small cell lung cancer, gastric cancer, differentiated thyroid cancer and ovarian cancer, especially CXCL8-251 A/T (Rafrafi et al., 2013; Koensgen et al., 2015; Kilic et al., 2016; Boonyanugomol et al., 2019). One of the most remarkable characteristics of CXCL8 is the variation of its expression levels. Normally, CXCL8 is undetectable in noninduced cells (Hoffmann et al., 2002). Mechanismly, in these unstimulated cells, the promoter of CXCL8 gene is repressed by three events: firstly, NF-κB-repressing factor (NRF) binds to the negative regulatory element (NRE) which overlaps the NF-κB binding site (Nourbakhsh et al., 2001); secondly, octamer-1 (OCT-1) binds to the complementary strand of the CXCL8 gene promoter in the opposite direction of the C/EBP site (Wu et al., 1997); and thirdly, deacetylation of the histone protein by histone deacetylase 1 (HDAC-1) (Ashburner et al., 2001). However, its expression is rapidly induced by various stimuli including cytokines such as IL-1 or TNFα, viral products or bacterials, other environmental stresses and transcription factors (including activator protein-1 (AP-1) and NF-κB) (Hoffmann et al., 2002; Helen et al., 2017). Remarkably, these stimuli cause a 5–100 folds increasing in CXCL8 expression(Hoffmann et al., 2002; Helen et al., 2017). Maximal CXCL8 expression and secretion is generated at least by a combination of three different mechanisms: 1) derepression of CXCL8 gene promoter; 2) transcriptional activation of CXCL8 gene by NF-κB and JNK pathways; 3) stabilization of CXCL8 mRNA by the p38 mitogen-activated protein kinase (MAPK) pathway (Hoffmann et al., 2002; Helen et al., 2017). The stability of the CXCL8 mRNA also plays an impact role on the secretion of CXCL8. In terms of mechanism, dual specificity mitogen-activated protein kinase kinase 6 (MKK6) by selectively activating p38 MAPK to activates MAP kinase-activated protein kinase 2 (MK2) which helps the stability of the CXCL8 mRNA (Helen et al., 2017; Hoffmann, et al., 2002). Additionally, in intestinal epithelial cells, carnosine could inhibit the translation of CXCL8 mRNA by phosphorylation of eIF4E (Son et al., 2008). Post-translational modification (PTM) of chemokines is an important mechanism of fine-tuning chemokine secretion, activation and selection of receptor (Vanheule et al., 2018). N-terminal shortening always associated with significantly increasing biological activity and receptor affinity of CXCL8. Numerous studies indicate that N-terminal truncation of CXCL8 (2 to 9–77) by CD13, CD26, MMP and so on (Van den Steen et al., 2003; Mortier et al., 2011; Vanheule et al., 2018). On the other side, citrullination of CXCL8 by peptidylarginine deiminase (PAD) could impair the effect of CXCL8 (Proost et al., 2008; Loos et al., 2009).

## Receptors of CXCL8: CXCR1 and CXCR2

**FIGURE 1:**
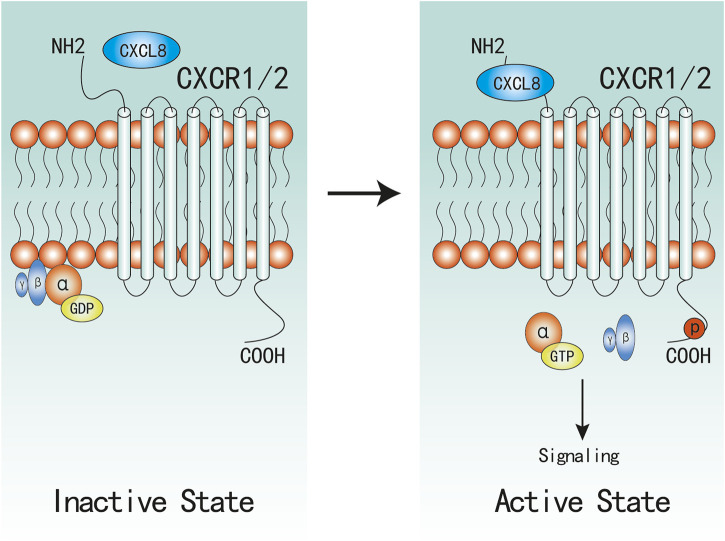
Active and Inactive Structures of CXCR1/2 Complex. CXCR1/2 is in complex with Gαβγ in inactive state. When CXCL8 binds to the N-terminal of CXCR1/2, α-GDP changes into α-GTP, and dissociates with βγ subunites, which would subsequently active associated signaling.

The receptors that bind to CXCL8 are two G protein coupled receptors (GPCR): CXCR1 and CXCR2 (Figure 1). The two receptors that are both the ELR + CXC receptors sharing 78% sequence homology between each other (Holmes et al., 1991; Murphy and Tiffany, 1991). CXCR1 and CXCR2 show different affinity to the different complexes of CXCL8. Both monomer and dimer forms of CXCL8 show similar affinity to CXCR2, however, only CXCL8 monomer functions as a potent CXCR1 agonist (Nasser et al., 2009; Das et al., 2010; Berkamp et al., 2017).

Upon CXCL8 binding, the following stable complexes will be formed: CXCL8(monomer)-CXCR1/2-G protein and CXCL8(dimer)-CXCR2-G protein (Park et al., 2012; Liu et al., 2020), which induce a conformational change of CXCR1/2 and facilitate the initiation of activation. Then, CXCR1/2 dissociate with the heterotrimeric G protein and then release the βγ subunites from the α subunite, which promotes the activation of several downstream signaling cascades (Waugh and Wilson, 2008; Liu et al., 2016). Similar to most GPCR, CXCR1/2 can also become phosphorylated, desensitized, and internalized upon binding to CXCL8. Despite evidenced that CXCR1/2 display similar downstream pathways, there remain marked differences between CXCR1 and CXCR2 in activation and signaling cascades. CXCR2 internalization occurs more rapidly and at lower ligand concentrations than CXCR1, and CXCR2 is also recycled back to the surface at a much slower rate than CXCR1 (Alfaro et al., 2017; Helen et al., 2017), which might be one possible mechanism that CXCR1 not CXCR2 can activate PLD (Stillie et al., 2009; Raghuwanshi et al., 2012; Cheng et al., 2019).

### Signaling Pathways of CXCL8-CXCR1/2 Axis

**FIGURE 2:**
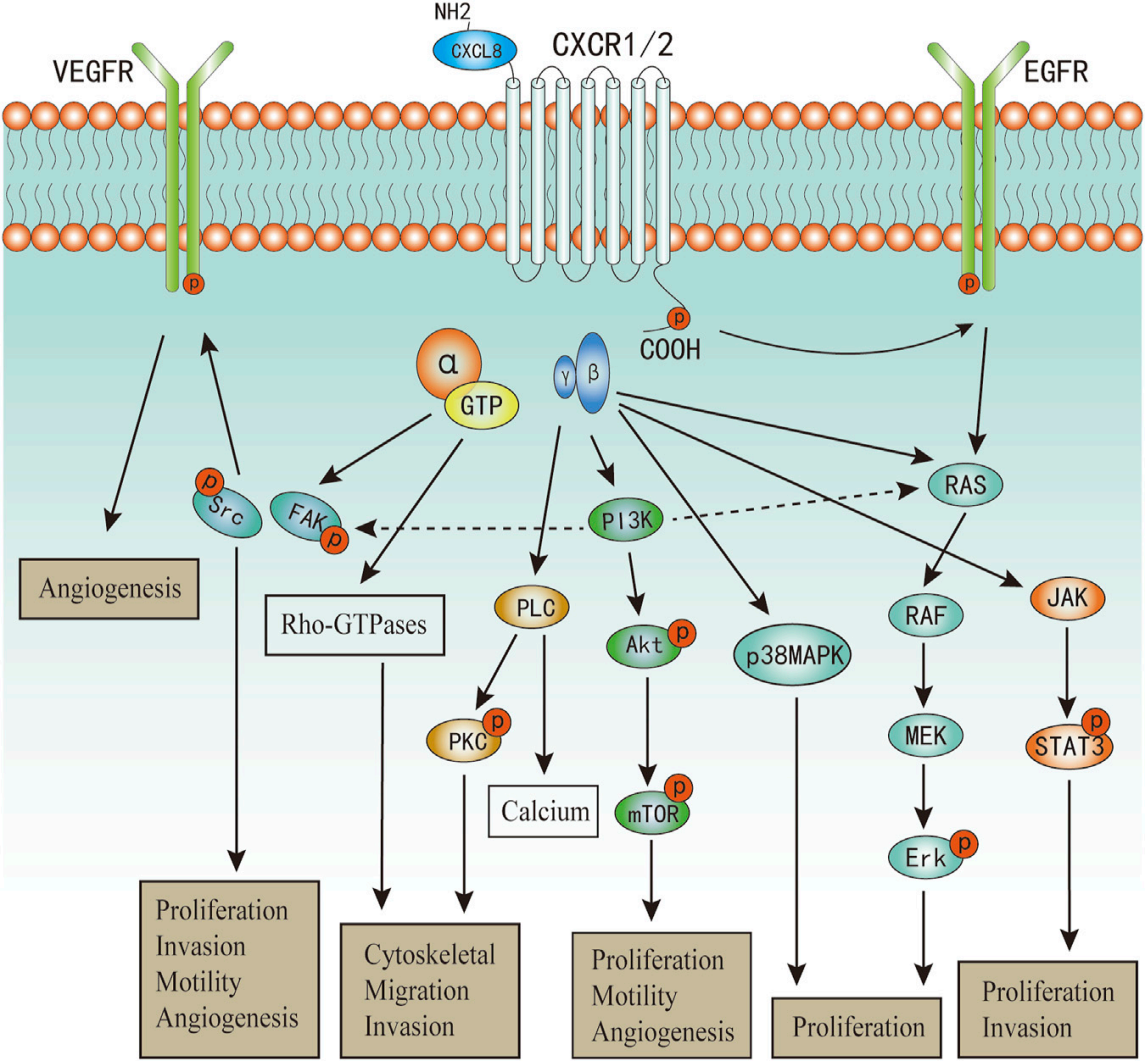
The major signaling pathways of CXCL8 in cancers.

Upon CXCL8 binding, CXCR1/2 can active multiple G-protein-mediated signalling cascades (Figure 2). Phosphatidylinositol-3 kinase (PI3K)/Akt is one of the principal downstream signal of CXCL8, which plays vital roles in modulating tumor motility, angiogenesis, and survival (Wilson et al., 2006; Waugh and Wilson, 2008). In androgen-independent prostate cancer, CXCL8 can also increase the expression of Akt (MacManus et al., 2007). PI3K can also act as an intermediate in coulping CXCR1/2 to MAPK and focal adhesion kinase (FAK)-Src signaling cascades (Knall et al., 1996; Waugh and Wilson, 2008). Phosphorylation of CXCR1/2 can also lead to two MyD88-dependent MAPK pathway: Erk-MAPK and p38 MAPK (Knall et al., 1997; Zhang et al., 2017; Cheng et al., 2019). Further, the Erk-MAPK cascades can be activated in indirect ways. Transactivation of epidermal growth factor receptor (EGFR) has been shown to occur in response to ligands of various GPCRs (Daub et al., 1996). The binding of CXCL8 to CXCR2 has been demonstrated to transactivate the EGFR resulting in Ras-GTPase activation, and subsequently actives the Erk-MAPK signaling cascades (Venkatakrishnan et al., 2000; Luppi et al., 2007). Activation of phospholipase C (PLC) by CXCL8 has been characterized in neutrophils and multiple cancer cells (Waugh and Wilson, 2008). The activated protein kinase C (PKC) can phosphorylate many cytoskeletal proteins that trigger dynamic alternations, facilitate cell adhesion and migration (Larsson, 2006; Quann et al., 2011). PKC can be classified into three categories (Larsson, 2006), and CXCL8 can active all the three categories of PKC mediated by PLC (Waugh and Wilson, 2008; Alassaf and Mueller, 2020). As a result of promoting these upstream signaling pathways, including PI3K/Akt, MAPK, and PLC/PKC, the activation of numerous transcription factors would be induced, one of which was nuclear factor-κB (NF-κB) (Waugh and Wilson, 2008; Gales, et al., 2013). Besides, activation of NF-κB is also one of the main mechanism to promote CXCL8 expression and secretion (Hoffmann, et al., 2002; Gales, et al., 2013; Helen, et al., 2017). Therefore, there exist a positive feedback between CXCL8 secretion and NF-κB activation, which has also been well described in a previous review (Gales, et al., 2013). Additionally, Numerous studies have confirmed that CXCL8 can induce the phosphorylation of protein tyrosine kinases, including FAK and Src kinases (Waugh and Wilson, 2008; Liu et al., 2016; Ju et al., 2017; Mohamed et al., 2020). Activation of FAK and Src kinases has been uncovered to promote cell proliferation, invasion, survival, and motility (Sulzmaier et al., 2014; Roskoski, 2015). In endothelial cells, it has revealed that CXCL8 can induce vascular endothelial growth factor receptor-2 (VEGFR2) phosphorylation mediated by the activation of Src kinases (Petreaca et al., 2007). CXCL8 can also promote dynamic and time-dependent induction of Rho-GTPases family in prostate cancer and endothelial cells (Schraufstatter et al., 2001; Waugh and Wilson, 2008; Yan et al., 2016). Recently, increased studies evidence that CXCL8 can induce the activation of Janus kinases and signal transducer and activator of transcription protein 3 (JAK/STAT3) signaling in both cancer and immune cells (Fu et al., 2015; Guo et al., 2017; Wu et al., 2019; Hu et al., 2020). Wu et al. demonstrated that CXCL8 could impair the function of NK cells by promoting STAT3 (Wu et al., 2019).

### Roles of CXCL8 in Tumor Biology

**FIGURE 3:**
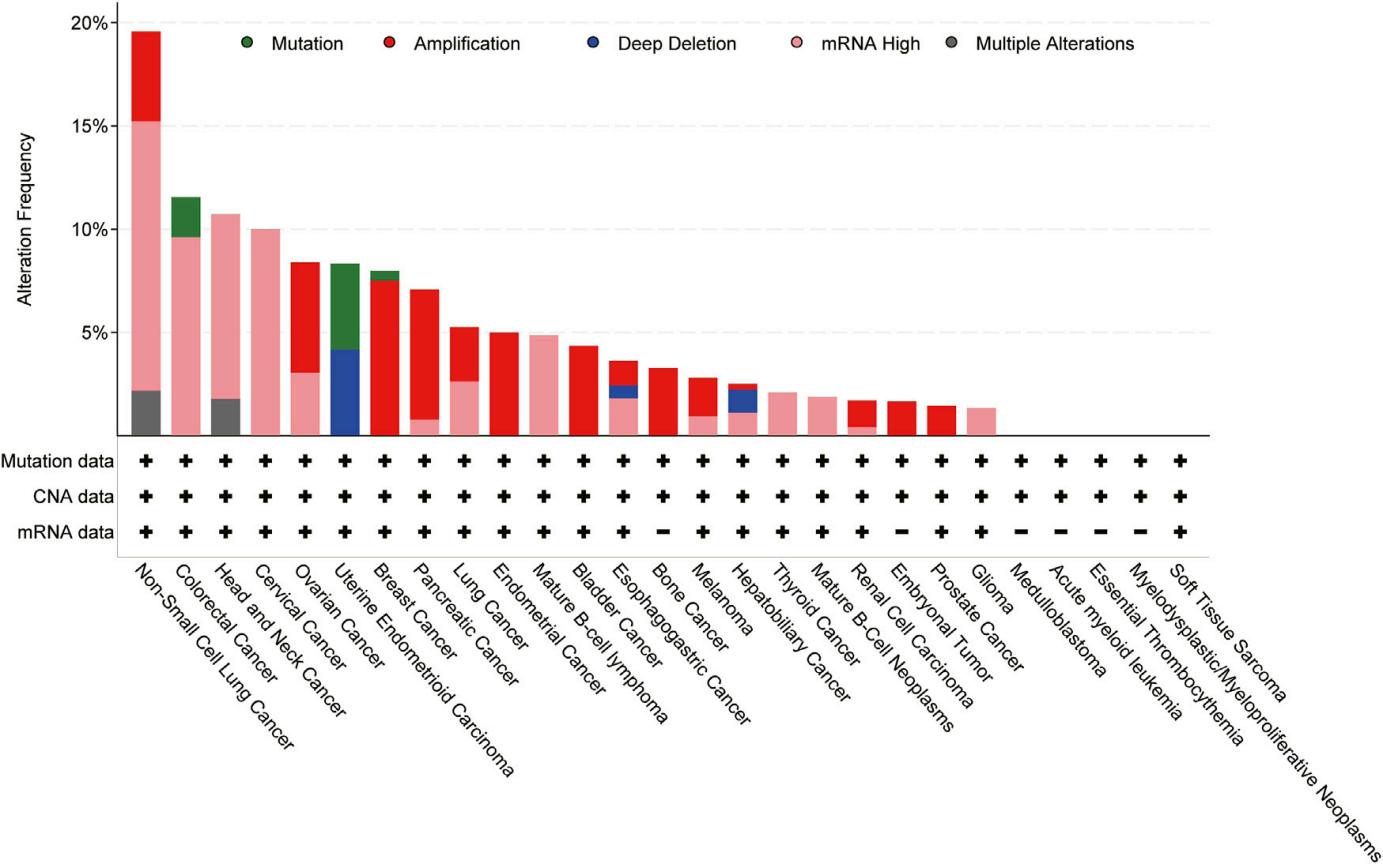
Genomic alterations of CXCL8 cross 27 cancer types; TCGA pan-cancer cohort from cBioPortal for Cancer Genomics were used for this analysis.

**FIGURE 4:**
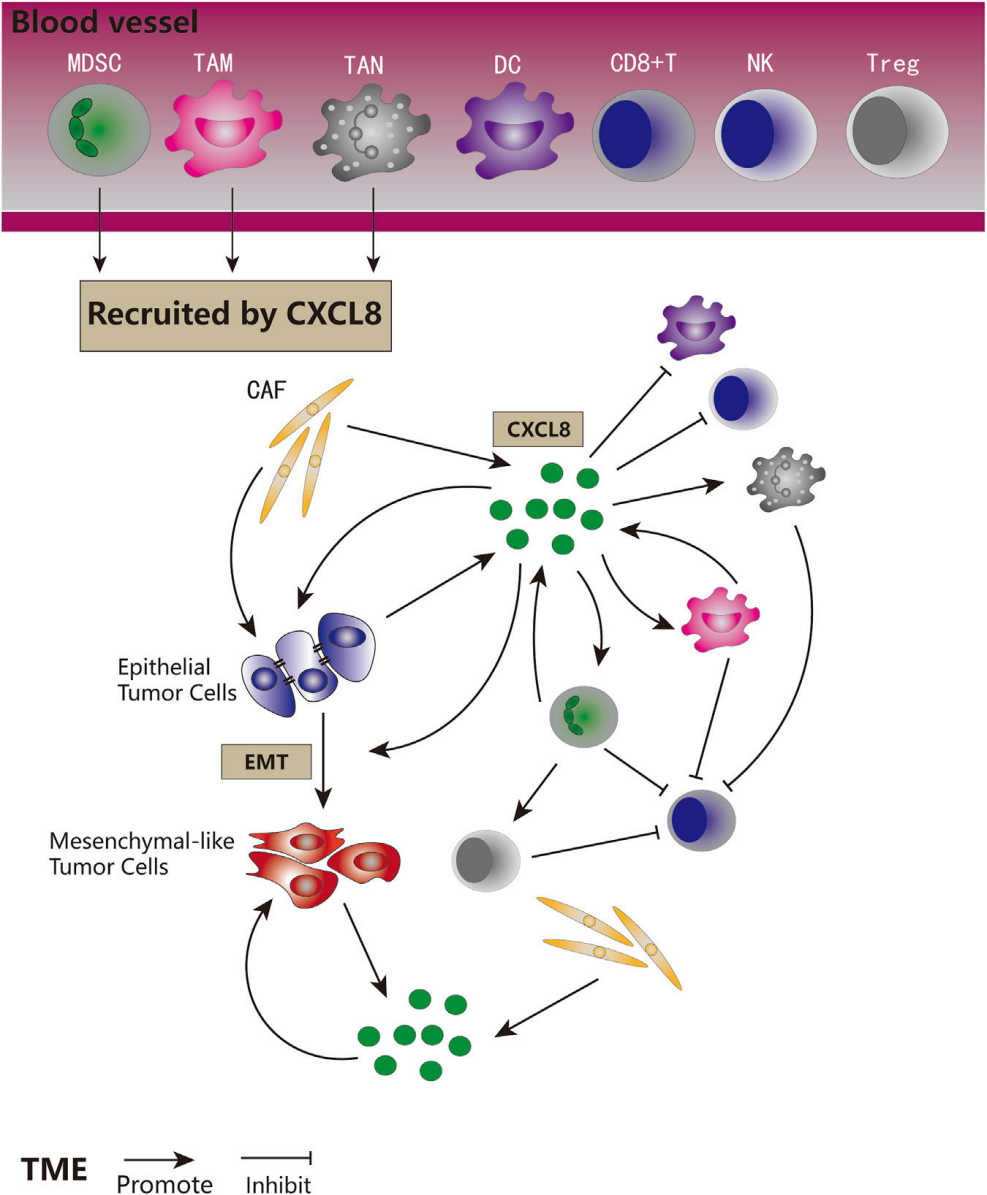
The role of CXCL8 signaling in tumor biology. CXCL8 recruited MDSCs, TAMs, and TANs to the TME. CXCL8 could be secreted by tumor cells, CAFs, MDSCs, and TAMs. CXCL8 could promote tumor cells proliferation and EMT, directly and indirectly. CXCL8 could facilitate the accumulation of pro-tumorigenic immune cells and tumor immune suppression, and inhibit anti-tumor immune cells in direct and indirect ways.

Expression of CXCL8 is significantly higher in numerous types of cancers (Figure 3), and many studies have evidenced that serum level of CXCL8 in patients with cancer can act as a prognostic marker (Cheng et al., 2019; Fousek et al., 2021). CXCL8 can promote tumor proliferation, survival, invasion, angiogenesis, tumor stemness and suppress anti-tumor immunity in direct and indirect manner (Figure 4) (Table 1). Growing evidence indicates that CXCL8 can directly contribute to the development of resistance to chemotherapy, molecularly targeted therapy, and immune checkpoint inhibition (ICI) therapy (Alfaro et al., 2017; Fousek et al., 2021). Therefore, CXCL8 has already been described as a pro-tumorigenic chemokine by impacting cancer cells and modifying TME to promote tumor progression and metastasis.

**TABLE 1:** The role of CXCL8 in common cancers. CSCs=Cancer Stem Cells

Cancer type	Function
Breast Cancer	Proliferation, Invasion and Migration, Angiogenesis, CSCs, Tumor Immune Suppression
Prostate Cancer	Proliferation, Invasion and Migration, Angiogenesis, CSCs, Tumor Immune Suppression
Lung Cancer	Proliferation, Angiogenesis, CSCs, Tumor Immune Suppression
Colon Cancer	Proliferation, Invasion and Migration, Angiogenesis, CSCs
Head and Neck Squamous Cell Carcinoma	Proliferation, Invasion and Migration
Osteosarcoma	Invasion and Migration
Glioma	Invasion and Migration, CSCs
Clear Cell Renal Cell Carcinoma	CSCs
Bladder Cancer	CSCs
Esophageal Carcinoma	CSCs
Hepatocellular Carcinoma	CSCs
Melanoma	Proliferation, Invasion and Migration, Angiogenesis, Tumor Immune Suppression
Ovarian Cancer	Proliferation, Invasion and Migration, Angiogenesis, Tumor Immune Suppression
Diffuse Large B-Cell Lymphoma	Tumor Immune Suppression
Pancreatic Cancer	Proliferation, Invasion and Migration, Angiogenesis, Tumor Immune Suppression
Gastric Cancer	Proliferation, Invasion and Migration, Angiogenesis, Tumor Immune Suppression

### Promoting Tumor Cells Proliferation and Survival by Novel Mechanisms

Many studies have proved that CXCL8 can promote cell proliferation and inhibit apoptosis in multiple cancers, including breast cancer, prostate cancer, lung cancer, colon cancer and so on (Liu et al., 2016). CXCL8 can mediate cancer cell proliferation both in autocrine and paracrine manner. Previous review has demonstrated that CXCL8 could be secreted by tumor cells and subsequently promote themselves growth and/or inhibit apoptosis (Liu et al., 2016). Some recent researches again proved this mechanism (Guo et al., 2017; Cui et al., 2018; Jia et al., 2018; Kumar et al., 2019).

Recently, increased evidence highlighted several novel mechanisms. As metabolism reprogramming has already become a hallmark of cancer, Xu et al. illustrate that CXCL8 could mediate enhancement of aerobic glycolysis in colorectal cancer (CRC) cells and reduce intracellular reactive oxygen species (ROS) levels, which subsequently promote CRC cell proliferation and invasion (Xu et al., 2017). We have also shown that CXCL8 can reduce the level of intracellular ROS by inhibiting the function of GSK-3β to suppress prostate cancer cell apoptosis (Sun L. et al., 2019). Components of TME play a vital role in progression and metastasis of cancer and can induce an upregulated cytokines and chemokines, such as CXCL8. Two studies conducted in CRC and pancreatic ductal adenocarcinoma indicated that mesenchymal stem cells (MSCs) and cancer-associated fibroblasts (CAFs) could promote cancer cells secreting CXCL8, then enhancing the ability of proliferation and invasion (Wang et al., 2015; Awaji et al., 2019). Furthermore, Yang et al. have demonstrated that there exists a positive feedback between CRC and neutrophil extracellular traps (NETs) mediated by CXCL8 (Yang L. et al., 2020). In addition to malignant cells, cells in TME can also secret CXCL8 and promote cancer cell proliferation, which can be supported by a recent study that suggested that CAFs in TME can release CXCL8 to increase the proliferation ability of gallbladder cancer cells (Chen et al., 2020).

### Promoting Tumor Cells Invasion and Migration by Novel Mechanisms

One of the main mechanisms used by tumor cells to obtain invasiveness and motility is the epithelial-to-mesenchymal transition (EMT). Present studies have proved that CXCL8 is essential for tumor cells to acquire and maintain this aggressive phenotype (Long et al., 2016; Fousek et al., 2021). As displayed above, components of TME can secret or promote cancer cells to secret CXCL8 which can also subsequently mediate the EMT of tumor cells. Further, there also exists an autocrine positive feedback loop between EMT and CXCL8 (David et al., 2016; Long et al., 2016). Many published researches indicate the important role of EMT in tumor resistance to chemotherapy, molecularly targeted therapy, and immune checkpoint inhibition (ICI) therapy (Horn et al., 2020b). Additionally, there are many excellent reviews about other mechanisms of CXCL8 in tumor therapy resistance (Alfaro et al., 2017; Cheng et al., 2019; Horn et al., 2020a; Fousek et al., 2021).

Emerging investigations have highlighted several novel mechanisms that are associated with the role of CXCL8 in cancer cell invasion and migration, including tumor heterogeneity, formation of feedback loop, and interacting with TME. Tumor heterogeneity is a vital feature of cancers, and cell sub-populations may interact with others to facilitate tumor progression (Meacham and Morrison, 2013). In the context of CRC, both hypoxic and fusobacterium nucleatum infected cancer cells can secret CXCL8 which subsequently contribute to the EMT of normoxic and noninfected cancer cells (Casasanta et al., 2020; Mi et al., 2020). Maynard et al. also demonstrate that only part of prostate cancer cells express CXCL8 in prostate cancer tissue microarrays, and high level of CXCL8 is associated with a more aggressive disease (Maynard et al., 2020). Pro-tumor feedback loops mediated by CXCL8 has been observed in multiple types of cancers. Xu et al. reported an intracellular feedback loop between CXCL8 and PTEN in HNSCC (Xu et al., 2020). Similarly, PTEN loss can also selectively upregulate the CXCL8 signaling in prostate cancer cells (Maxwell et al., 2013). Effects of CXCL8 on tumor cells could also influence the TME or be influenced by the components of TME. MSCs and NET can also form a positive pro-tumor feedback loop with osteosarcoma and glioma cells *via* CXCL8, respectively (Kawano et al., 2018; Zha et al., 2020). Previous reviews and many recent studies have illustrated that multiple cell types in TME can directly secret CXCL8, or regulate the expression of CXCL8 in cancer cells, or be regulated by CXCL8 derived from cancer cells to promote tumor invasion and migration (Zheng et al., 2018; Cheng et al., 2019; Nie et al., 2019; Fousek et al., 2021).

### Promoting Tumorogenic Angiogenesis

Angiogenesis has been recognized as a hallmark of cancer, which is necessary for tumor survival and disseminating to a new location (Hanahan and Weinberg, 2011). Effect of CXCL8 on tumor angiogenesis has been widely investigated, and CXCL8 has already be defined as a pro-angiogenesis chemokine (Liu et al., 2016; Cheng et al., 2019; Fousek et al., 2021; Ueda et al., 2022). Human vascular endothelial cells constitutively express CXCR2 (Cheng et al., 2019). Upon cancer cells and some types of stroma cells secreting CXCL8 in TME, endothelial cells begin to express and secret matrix metalloproteinases (MMPs) to break down the extracellular matrix (ECM), then, resulting in angiogenesis (Li et al., 2003). In addition, CXCL8 can also induce recruitment of endothelial cells which participate directly in vascularization (Strieter et al., 1995). Intriguingly, there is also a crosstalk between CXCL8 and VEGFR2 in angiogenesis (Petreaca et al., 2007). In this context, a loop in endothelial cells have been discovered, which is that CXCL8 can increase the secretion of VEGF-A and induce the expression of VEGFR2 in endothelial cells (Martin et al., 2009; Alfaro et al., 2017).

### Promoting Development of Cancer Stem Cells

A plethora of literature indicates that CXCL8 is involved in the maintenance of cancer stem cells (CSCs) which is always associated with tumor development and progression, treatment resistance and used to explain heterogeneity in solid tumors (Raza et al., 2022). Generally, the CXCL8-CXCR1/2 axis plays impact roles on formation, development or invasion of CSCs in colon cancer (Luo et al., 2018; Fisher et al., 2019; Kim et al., 2021), breast cancer (Choi et al., 2009), glioblastoma (Zhou et al., 2014; McCoy et al., 2019), clear cell renal cell carcinoma (ccRCC) (Corrò et al., 2019), pancreatic cancer (Chen et al., 2014), hepatocellular carcinoma (HCC) (Kahraman et al., 2019), lung cancer (Shimizu and Tanaka, 2019), bladder cancer (Zhou et al., 2021) and esophageal carcinoma (Huang et al., 2017). Furthermore, CXCL8 has also been found to promote the interaction between CSCs and mesenchymal stem cells (MSCs) to further enlarging the population of CSCs in colon cancer (Ma et al., 2020; Ma et al., 2021). Given the crucial role of CXCL8 in CSCs, targeting CXCL8-CXCR1/2 axis as a component of combination therapy has also been explored. Pre-clinical studies demonstrate that combining CXCR1/2 inhibitors with the human epidermal growth factor receptor 2 (HER2)-targeted therapies has potential as an effective treatment strategy to repress CSCs activity in breast cancer (Singh et al., 2013). In HCC, after inhibiting CXCR1/2 by Reparixin or knockdown CXCL8, CSCs features of HCC were reduced, and sensitivity to Sorafenib increased significantly (Kahraman et al., 2019).

### Inhibiting Anti-Tumor Immunity

CXCL8 also has potent ability on modulating immune cell chemotaxis and functions. CXCL8 derived from tumor can act in a paracrine manner to change the composition of immune infiltration in TME, resulting the accumulation of pro-tumorigenic immune cells and tumor immune suppression (Alfaro et al., 2017; Horn et al., 2020b). Published reports collectively suggest that CXCL8 can recruit tumor-associated macrophages (TAMs), myeloid derived suppressor cells (MDSCs), and neutrophils to the TME, resulting in dampening the anti-tumor immune response of cytotoxic immune cells. Correspondingly, it can also attenuate the anti-tumor activity of dendritic cells (DCs) and NK cells (Alfaro et al., 2017; Fousek et al., 2021).

Neutrophils make up a sizeable part of immune cells in TME, so-called tumor-associated neutrophils (TANs), which can be divided into antitumor N1 and pro-tumor N2 phenotypes (Fridlender et al., 2009). Further, the N2 TANs promote tumor progression by activating tumor angiogenesis, suppressing the function of anti-tumor T cells, and recruiting T regulatory cells (Tregs) (Rodriguez et al., 2004; Nozawa et al., 2006; Mishalian et al., 2014; David et al., 2016). Unfortunately, there are no groups directly investigating the relationship between CXCL8 and N2 phenotype. Several recent reports preliminary demonstrate the effect of CXCL8 on TANs. In ovarian cancer, CXCL8 can recruit TANs in TME and induce the expression of Jagged2 (JAG2) in TANs which subsequently inhibit the activity of CD8(+) T cells (Yang M. et al., 2020). Additionally, TANs in the TME of diffuse large B-cell lymphoma (DLBCL) can also increase the secretion of april mediated by CXCL8, and promote DLBCL progression (Manfroi et al., 2017).

MDSCs are known as a heterogeneous population of immature immunosuppressive cells, which derive from myeloid progenitor cells, accumulate in the circulation and the TME of most cancer patients (Gabrilovich and Nagaraj, 2009; David et al., 2016). Generally, MDSCs can be divided into two subtypes: granulocytic (CD33 ^+^ CD11b + HLA-DR-/low CD15^+^, PMN-MDSC) or monocytic (CD33 + CD11b + HLA-DR-/low CD14^+^, M-MDSC) (Poschke and Kiessling, 2012). In TME, MDSCs have been shown to act as a driver of immune suppression by inactivating T cell receptors, starving T cell, inhibiting T cell proliferation, recruiting CAFs, and inducing Tregs (David et al., 2016; Horn et al., 2020a). Increasing investigations evidence the role of CXCL8 in attracting and enhancing the function of MDSCs which then attenuate the activation of CD8(+) T cell (Asfaha et al., 2013; Katoh et al., 2013; Dominguez et al., 2017; Najjar et al., 2017; Li et al., 2018). Interestingly, CXCL8 can recruit the both subtypes of MDSCs to TME, but they play different functions (Alfaro et al., 2016). The M-MDSCs can directly suppress the activity of T cells, however, the PMN-MDSC subset only induce the formation of NETs which might facilitate tumor cell migration. Further, a recent report indicates a novel subtype of MDSCs that attract by CXCL8 in human gastric cancer (Mao et al., 2018). After attracting CD45(+)CD33(low)CD11b(dim) MDSCs to TME, CXCL8 will promote this novel subset MDSCs expressing arginase I that contributes to CD8(+) T cell suppression via PI3K/Akt signaling. Supportively, in prostate cancer and melanoma, the increasing level of circulating MDSCs are evidenced to be associated poor clinical outcomes and high plasma CXCL8 concentration (Chi et al., 2014; Tobin et al., 2019).

Tumor-associated macrophages (TAMs) are present in all stages of tumors and exert a dual effectiveness on tumor progression (Noy and Pollard, 2014; Mantovani et al., 2017). Similar to TANs, TAMs have been divided into two subsets: antitumor M1-like and tumor-promoting M2-like phenotype (Noy and Pollard, 2014). Numerous studies have concentrated on the role of TAMs on CXCL8, which have indicated that TAMs can express or induce tumor cell secreting CXCL8 to contribute to tumor progression (Chen et al., 2003; Chen et al., 2018; Zheng et al., 2018). Emerging reports uncover that CXCL8 also have vital roles in recruiting and directing the polarization of TAMs. The findings of Zhang and colleagues suggest that the CXCL8-CXCR2 axis can promote trafficking of CXCR2(+) CD68(+) macrophages to pancreatic cancer TME, and the recruitment TAMs can inhibit the efficacy of PD1 blockade (Zhang et al., 2020). Further investigation performed in gastric cancer indicates that TAMs can induce themselves increasing the expression of PD-L1 and decrease CD8(+) T cells infiltration by secreting CXCL8 (Lin et al., 2019). Meanwhile, CXCL8 also plays a role in inducing a shift in TAMs toward the M2 phenotype (Krawczyk et al., 2017; Ning et al., 2018).

DCs and NK cells play important roles in adaptive and innate anti-tumor immune response, respectively. Previous studies suggest that CXCL8 derived from tumor cells can disorients DCs migration without impairing the stimulation of T-cell (Feijoó et al., 2005; Alfaro et al., 2011; Li et al., 2021). A recent investigations indicates that tumor cells can secret CXCL8 to impair the functions of NK cells via STAT3 signaling (Wu et al., 2019).

### CXCL8 in Immune Checkpoint Therapy

Many excellent reviews demonstrate the role of CXCL8-CXCR1/2 axis in target therapy, chemotherapy and the prognostic role of plasma level of CXCL8 in cancer (Liu et al., 2016; Alfaro et al., 2017; Cheng et al., 2019; Fousek et al., 2021). Here, we highlight current preclinical and clinical studies correlating CXCL8 to immunotherapy. Immune checkpoint inhibitions (ICIs) has become the cornerstone of immunotherapy in many types of cancers (Ribas and Wolchok, 2018; Bakouny and Choueiri, 2020). However, not all cancer patients have a good response to ICIs, and early determining the clear group which is sensitive or resistant to ICIs can improve clinical outcomes. Recent evidences suggest that CXCL8 plays an important role in response quality on ICIs.

A small retrospective trial suggests that increasing level of serum CXCL8 can predict resistant to anti-PD-1 treatment in non-small-cell lung cancer (NSCLC) patients (Sanmamed et al., 2017). To further investigating the role of CXCL8 in predicting response of patients treated with ICIs, two recent trials with large sample size have been performed to evaluate the correlation between plasma CXCL8 and cancer progression (Schalper et al., 2020; Yuen et al., 2020). Schalper et al., using a large cohort with 1,344 patients, show that high baseline plasma CXCL8 level is associated with poor clinical outcomes in participants with advanced melanoma, NSCLC, and renal-cell carcinoma (RCC) treated with nivolumab or ipilimumab, everolimus or docetaxel, which indicate that serum CXCL8 level is an unfavorable factor in tumor immunobiology and can act as an independently predictive biomarker in patients receiving ICIs (Schalper et al., 2020). Another multiple randomized trials with 1,445 patients in metastatic urothelial carcinoma (mUC) and mRCC confirms this finding (Yuen et al., 2020). Both trials further suggest that greater CXCL8 expression in tumor is associated with higher plasma CXCL8 level, an immunosuppressive myeloid-enriched TME, and T cell suppression (Schalper et al., 2020; Yuen et al., 2020). Additionally, Yuen and colleagues also further stratify patients using plasma CXCL8 level and T cell effector signature score. Patients with high T cell effector signature score and low plasma CXCL8 level can derive best benefit from ICIs (Yuen et al., 2020).

Emerging studies suggest that combination of targeting CXCL8-CXCR1/2 axis and ICIs can provide further benefit in anti-tumor efficacy. In the context of breast and lung cancer, combined SX-682, an bioavailable small-molecule inhibitor of CXCR1 and CXCR2, and anti-PD-1/PD-L1 can achieve best tumor control in murine model (Sun Y. et al., 2019; Horn et al., 2020b). Interestingly, both studies indicate that inhibition of CXCR1/2 eventually results in reducing infiltration with PMN-MDSCs which play a vital role in T cell suppression. Zhang et al. discover that treated with interferon gamma (IFN-γ) can suppress a variety of pancreatic cancer derived CXCL8 and tumor-derived CXCL8 deficiency inhibit the trafficking of M2 TAM (Zhang et al., 2020). Further, combined IFN-γ and anti-PD-1 treatment enhance the anti-tumor efficacy. As for directly targeting CXCL8, in triple-negative breast cancer (TNBC), HuMax-IL8, a fully human monoclonal antibody that inhibits CXCL8, can significantly reduce the infiltration of PMN-MDSCs to TME and enhance the efficacy of immunotherapy (Dominguez et al., 2017).

Bilusic et al. conduct a phase I clinical trial including 15 patients with metastatic or unresectable solid tumors treated with HuMax-IL8 (Bilusic et al., 2019). The results of this trial indicate that HuMax-IL8 is safe and well-tolerated. This trial could be a basic evidence for further evaluating the combination of CXCL8 blockade and other immunotherapies. In addition, several ongoing studies have been designed to evaluate the safety and efficacy of combined HuMax-IL8 and immunotherapy in cancer patients (Table 2).

**TABLE 2:** Clinical Studies of combination of ICI agents and CXCL8 blockade. A = active; R = recruiting.

Checkpoint agent	CXCL8 blockade	Cancer type	Sample size	Clinical trial number	Status
Nivolumab	HuMax-IL8	Advanced Cancers	320	NCT03400332	A
Nivolumab	HuMax-IL8	Hepatocellular Carcinoma	74	NCT04050462	R
Nivolumab	HuMax-IL8	Hormone-Sensitive Prostate Cancer	60	NCT03689699	R

### Future Directions

Expression of CXCL8 is significantly higher in numerous types of cancers, and high expression of CXCL8 is significantly associated with shorter median overall survival in many kinds of cancers based on TCGA database (Table 3). The findings of numerous researches involving preclinical *in vitro* and *in vivo* models illustrate that combination of targeting CXCL8-CXCR1/2 axis and ICIs can provide further benefit in anti-tumor efficacy. In addition, clinical trials demonstrate that CXCL8 plays an important role in response quality on ICIs. Furthermore, activation of CXCL8-CXCR1/2 axis and its downstream signaling pathways play important roles in tumor survival and invasion, and suppress antitumor immune responses in the TME. Therefore, therapies targeting this axis are likely to benefit patients with cancer by inhibiting tumor growth and stimulating antitumor immunity. Clinical trials are ongoing to prove this.

**TABLE 3:** The correlation between CXCL8 expression and cancer survival in TCGA cohorts. HR, hazard ratio; CI, confidence interval; OS, overall survival; NA, not analysis.

Cancer type	HR(95%CI)	Median OS (Months)	
		Low expression cohort	High expression cohort
Cervical squamous cell carcinoma	2.97 (1.78, 4.94)	68.40	25.77
Esophageal Adenocarcinoma	2.76 (1.40, 5.54)	46.73	9.07
Head-neck squamous cell carcinoma	1.54 (1.15, 2.07)	58.27	33.27
Kidney renal clear cell carcinoma	1.89 (1.37, 2.61)	55.23	31.53
Kidney renal papillary cell carcinoma	2.44 (1.03, 5.77)	NA	NA
Liver hepatocellular carcinoma	2.40 (1.60, 3.59)	104.17	45.73
Lung adenocarcinoma	1.58 (1.09, 2.29)	59.27	42.93
Lung squamous cell carcinoma	1.38 (1.05, 1.81)	63.73	38.47
Pancreatic ductal adenocarcinoma	2.10 (1.23, 3.58)	72.73	19.73
Sarcoma	1.66 (1.09, 2.50)	89.80	54.97
Stomach adenocarcinoma	1.49 (1.06, 2.10)	43.13	22.50
Thyroid carcinoma	3.29 (1.18, 9.17)	NA	NA

As metabolism reprogramming has already become a hallmark of cancer, the relationship between CXCL8 and tumor metabolism reprogramming is not widely explored. How CXCL8 influences tumor metabolism and subsequently facilitate cancer proliferation needing further investigation. In addition, present studies demonstrated that part of CRC cells could secreted CXCL8 which contributed to the EMT of the remaining cells not secreting CXCL8. However, the mechanism of this phenomenon is not clear. Further exploration is needed to determine if other types of cancer exist similar phenomenon. As mentioned, CXCL8 can recruit TANs in TME. Unfortunately, there are no groups directly investigating the relationship between CXCL8 and N2 phenotype. Meanwhile, CXCL8 also plays a role in inducing a shift in TAMs toward the M2 phenotype. However, the mechanism is unclear.

## Conclusion

The Chemokine CXCL8 is well accepted to play a crucial role in tumor survival, invasion, and TME angiogenesis, immune suppression via several types of intracellular signaling pathway. To date, many novel mechanisms to mediate the above tumor biology by CXCL8 have been highlighted. More and more evidences indicate that CXCL8 should be considered as a pro-tumor factor with dual roles: directly promoting tumor survival and affecting components of TME to indirectly facilitate tumor progression. Further, CXCL8 can also act as an important predictor of the clinical outcomes for ICIs. As cancer therapy advances, some emerging clinical trials are ongoing to explore the efficacy and safety of combining anti-CXCL8 and ICIs therapy.
